# Aeromagnetic Compensation for UAVs Using Transformer Neural Networks

**DOI:** 10.3390/s25226852

**Published:** 2025-11-09

**Authors:** Weiming Dai, Changcheng Yang, Shuai Zhou

**Affiliations:** 1 College of Information Engineering, Gan Dong University, Fuzhou 344000, China; gdxy_dwm519@163.com; 2 College of Intelligent Robotics and Advanced Manufacturing, Fudan University, Shanghai 200433, China; 3 College of GeoExploration Science and Technology, Jilin University, Changchun 130026, China; yangcc22@mails.jlu.edu.cn

**Keywords:** aeromagnetic compensation, T-L model, transformer, MLP, neural networks

## Abstract

In geophysics, aeromagnetic surveying based on unmanned aerial vehicles (UAV) is a widely employed exploration technique, that can analyze underground structures by conducting data acquisition, processing, and inversion. This method is highly efficient and covers large areas, making it widely applicable in mineral exploration, oil and gas surveys, geological mapping, and engineering and environmental studies. However, during flight, interference from the aircraft’s engine, electronic systems, and metal structures introduces noise into the magnetic data. To ensure accuracy, mathematical models and calibration techniques are employed to eliminate these aircraft-induced magnetic interferences. This enhances measurement precision, ensuring the data faithfully reflect the magnetic characteristics of subsurface geological features. This study focuses on aeromagnetic data processing methods, conducting numerical simulations of magnetic interference for aeromagnetic surveys of UAVs with the Tolles–Lawson (T-L) model. Recognizing the temporal dependencies in aeromagnetic data, we propose a Transformer neural network algorithm for aeromagnetic compensation. The method is applied to both simulated and measured flight data, and its performance is compared with the classical Multilayer Perceptron neural networks (MLP). The results demonstrate that the Transformer neural networks achieve better fitting capability and higher compensation accuracy.

## 1. Introduction

Aeromagnetic surveying is a method that uses magnetometers in aircraft for rapid, large-scale detection in geophysics. Aeromagnetic measurement can obtain physical quantities that reflect changes in the Earth’s magnetic field. Through data processing, local magnetic anomalies caused by mineral deposits, oil and gas reservoirs, or geological structures can be identified. Aeromagnetic surveys offer advantages such as high data acquisition efficiency and high resolution, making them widely applicable in mineral resource exploration, oil and gas investigations, fundamental geological mapping, and military target detection [1,2].

However, during aeromagnetic surveying, the presence of ferromagnetic materials in the airborne platform, along with various influencing factors during flight, affects data acquisition, subsequently impacts post-acquisition processing and geological interpretation. Therefore, compensation of the measured magnetic data is required [3,4]. The earliest aeromagnetic compensation methods primarily relied on hardware-based approaches. Tolles and Lawson established the classic aeromagnetic interference model (T-L model). There are three components in the interference field: permanent field, induced field, and eddy currents field. They also established a model called the compensation matrix for compensation coefficients solving [5]. This laid the foundation for aeromagnetic compensation. With early methods often involving the installation of counteracting magnets or coils on the aircraft to physically cancel out the permanent interference of the airframe and eddy currents interference generated during flight. With advancements in computer and electronic technologies, aeromagnetic compensation gradually shifted toward software-based methods. The aeromagnetic interference field can establish the T-L linear equation regarding attitude variation based on the T-L model, and then obtain the compensation coefficient through optimization solution. However, solving the T-L equation suffers from multicollinearity issues. To address this problem, a small-signal method proposed by Bickel can obtain more stable solutions by weakening linear dependencies between variables [6]. The most classical approach is least squares, known as the T-L compensation matrix method. However, the traditional solution methods have certain multicollinearity in the solution of the T-L equation, and the calculation of the compensation coefficient has a large calculation error. In order to improve the stability of the solution, Leach and Wu et al., respectively, adopted the ridge regression method and principal component analysis (PCA) to solve the aeromagnetic compensation coefficient [7,8].

The performance of drones employed in aeromagnetic surveys undoubtedly constitutes a critical factor affecting measurement data quality. The evolution of drone technology traces back to early 20th-century military target drone experiments. In 1916, American inventor Charles Kettering developed the “Kettering Bug,” an automatically controlled aerial bomb regarded as the prototype of modern drones [9]. During the 1930s, Britain created the “Queen Bee” target drone for anti-aircraft artillery training [10]. The Cold War witnessed rapid advancements in drone technology, with applications expanding into reconnaissance missions and tactical systems [11,12]. Since the 1990s, progress in GPS and composite materials technology drove the miniaturization and precision enhancement of drones [13]. The 21st century saw explosive growth in civilian drones, with French Parrot and Chinese DJI dominating the consumer market, while AI technology further catalyzed the development of autonomous flight systems [14,15,16]. With the development of artificial intelligence, drones are widely used in agriculture, remote sensing, communications, and other fields [17,18,19]. In the field of geophysics, a comprehensive overview of the applications of drone technology has been provided by Dimitris Perikleous et al. in geophysical exploration [20].

Since the 21st century, various intelligent algorithms have been applied to aeromagnetic compensation to further improve accuracy and adaptability in complex environments. Among these, neural network methods have been the most representative. In 1993, Williams pioneered a new aeromagnetic compensation by using neural networks, employing multi-layer backpropagation (BP) neural networks to model aircraft attitude variables, achieving compensation effects comparable to the traditional T-L model [21]. Ma et al. introduced unscented Kalman filtering into the solution of the T-L equation, effectively improving the compensation effect [22]. By 2022, Yu et al. introduced BP neural networks with residual connections to address gradient vanishing issues [23], while Jiao et al. achieved real-time compensation using backpropagation neural networks under limited computational power [24]. Additionally, Zhou et al. developed radial basis function neural networks (RBF-NN) to enhance generalization capability, thereby improving the precision of aeromagnetic compensation [25]. Although these methods improve the accuracy of aeromagnetic compensation to some extent, they do not take into account the time correlation of aeromagnetic data.

With the continuous development of deep neural networks, Transformer has been widely applied in fields such as time series prediction and natural language processing [26,27]. Since magnetic fields represent physical quantities that vary continuously over time, and optically pumped magnetometers typically measure magnetic fields at fixed sampling rates, their output signals inherently possess temporal characteristics [28,29]. To further enhance the accuracy of aeromagnetic compensation, this study employs Transformer neural networks for aeromagnetic compensation. The model effectively captures temporal dependencies, improves data fitting performance, and ultimately enhances compensation accuracy, as validated by both simulated and measured data.

## 2. Aeromagnetic Compensation Model and Methodology

### 2.1. Tolles and Lawson Model

**Figure 1:**
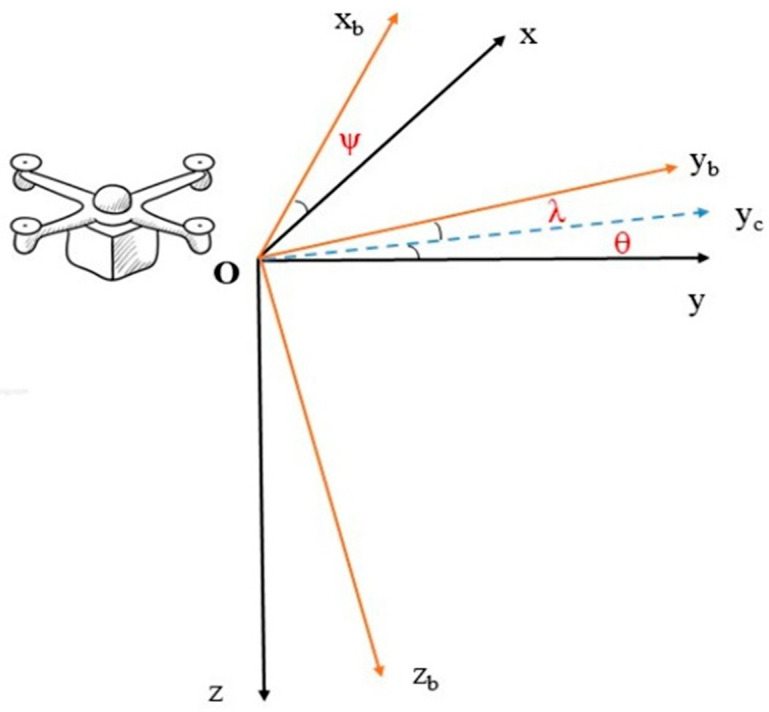
The transformation relationship of coordinate systems.

When establishing the T-L model, it is necessary to clarify the corresponding relationship between the aircraft coordinate system and the geographic coordinate system. As shown in Figure 1, the position of the coordinate origin is defined as the center of the aeromagnetic measurement system. The geographic coordinate system is shown in the x-y-z black color coordinate system in Figure 1, while the aircraft coordinate system is shown in the xb-yb-zb yellow color coordinate system in Figure 1. The attitude changes in the drones during flight can be divided into three basic motions: pitches, rolls, and yaws, specifically, pitch angle θ, roll angle λ, and yaw angle ψ.

Based on the mechanisms of magnetic interference generation, the aeromagnetic interference was classified into three categories. The permanent field originates from hard magnetic materials (such as permanent magnets) and direct current closed circuits on the aircraft. Its intensity and direction do not change with flight attitude, remaining constant for a given aircraft. The induced field is caused by paramagnetic or soft materials magnetized by the earth’s magnetic field. The eddy current field is a transient interference caused by the aircraft’s metal structure cutting geomagnetic flux lines during motion. Its strength and direction depend on the geomagnetic gradient, maneuvering actions, and flight acceleration [5].

These three types of interference in the aircraft coordinate system would be expressed as follows:

(1) $$H_{P}=c_{1}u_{1}+c_{2}u_{2}+c_{3}u_{3}$$

(2) $$H_{i}=T_{t}(c_{4}u_{1}^{2}+c_{5}u_{1}u_{2}+c_{6}u_{1}u_{3}+c_{7}u_{2}^{2}+c_{8}u_{2}u_{3}+c_{9}u_{3}^{2})$$

(3) $$\begin{array}{l} H_{ec}=T_{t}(c_{10}u_{1}u_{1}^{\prime }+c_{11}u_{2}u_{1}^{\prime }+c_{12}u_{3}u_{1}^{\prime }+c_{13}u_{1}u_{3}^{\prime }\\ +c_{14}u_{2}u_{3}^{\prime }+c_{15}u_{3}u_{3}^{\prime }+c_{16}u_{1}u_{2}^{\prime }+c_{17}u_{2}u_{2}^{\prime }+c_{18}u_{3}u_{2}^{\prime }) \end{array}$$

(4) $$u_{1}=\frac{T_{bx}}{T_{t}},\;u_{2}=\frac{T_{by}}{T_{t}},\;u_{3}=\frac{T_{bz}}{T_{t}}$$

(5) $$T_{t}=\sqrt{T_{bx}^{2}+T_{by}^{2}+T_{bz}^{2}}$$

where *T* is the intensity of the geomagnetic field, *c*_1_, *c*_2_, ……, *c*_18_ are the compensation coefficients to be determined, *u*_1_, *u*_2_, and *u*_3_ denote the direction cosines, $u_{1}^{\prime }$, $u_{2}^{\prime }$, $u_{3}^{\prime }$ represent derivatives of these cosines with respect to time *t*, the total geomagnetic field is *T_t_*, and *T_bx_*, *T_by_*, and *T_bz_* are the triaxial components of the fluxgate sensor. Due to the lower measurement accuracy of triaxial fluxgate magnetometers compared to optically pumped magnetometers, as well as installation misalignments introducing additional errors, the triaxial fluxgate data may exhibit deviations, thereby affecting compensation performance. Therefore, this paper indirectly calculates the three-component geomagnetic field data based on the normal geomagnetic field and attitude angle information:

(6) $$\left[\begin{array}{l} T_{bx}\\ T_{by}\\ T_{bz} \end{array}\right]=D\left[\begin{array}{l} T_{gx}\\ T_{gy}\\ T_{gz} \end{array}\right]$$

(7) $$D=\left[\begin{array}{ccc} \cos\theta \cos\psi & \sin\theta \cos\psi & -\sin\psi \\ \cos\theta \sin\lambda \sin\psi -\sin\theta \cos\lambda & \sin\theta \sin\lambda \sin\psi +\cos\theta \cos\lambda & \sin\lambda \cos\psi \\ \cos\theta \cos\lambda \sin\psi +\sin\theta \sin\lambda & \sin\theta \cos\lambda \sin\psi -\cos\theta \sin\lambda & \cos\lambda \cos\psi \end{array}\right]$$

(8) $$T_{gx}=T_{t}\cos\varphi \cos\mu$$

(9) $$T_{gy}=T_{t}\cos\varphi \sin\mu$$

(10) $$T_{gz}=T_{t}\cos\varphi \sin\varphi ,$$

where μ is the magnetization bias angle, and φ is the magnetization tilt angle.

According to the above formulas, the total interference magnetic field *H_t_* is expressed as follows:

(11) $$\begin{array}{ll} H_{t} & =H_{p}+H_{i}+H_{ec}\\ & =T_{t}(c_{1}u_{1}/T_{t}+c_{2}u_{2}/T_{t}+c_{3}u_{3}/T_{t}\\ & +c_{4}u_{1}^{2}+c_{5}u_{1}u_{2}+c_{6}u_{1}u_{3}+c_{7}u_{2}^{2}+c_{8}u_{2}u_{3}+c_{9}u_{3}^{2}\\ & +c_{10}u_{1}u_{1}^{\prime }+c_{11}u_{2}u_{1}^{\prime }+c_{12}u_{3}u_{1}^{\prime }+c_{13}u_{1}u_{3}^{\prime }+c_{14}u_{2}u_{3}^{\prime }\\ & +c_{15}u_{3}u_{3}^{\prime }+c_{16}u_{1}u_{2}^{\prime }+c_{17}u_{2}u_{2}^{\prime }+c_{18}u_{3}u^{\prime }) \end{array},$$

Moreover, since:

(12) $$\begin{array}{l} u_{1}^{2}+u_{2}^{2}+u_{3}^{2}=1\\ u_{1}u_{1}^{\prime }+u_{2}^{\prime }u_{2}^{\prime }+u_{3}u_{3}^{\prime }=0 \end{array},$$

we obtain:

(13) $$H_{t}={\left[\begin{array}{c} b_{1}\\ b_{2}\\ b_{3}\\ b_{4}\\ b_{5}\\ b_{6}\\ b_{7}\\ b_{8}\\ b_{9}\\ b_{10}\\ b_{11}\\ b_{12}\\ b_{13}\\ b_{14}\\ b_{15}\\ b_{16} \end{array}\right]}^{T}\left[\begin{array}{c} u_{1}/T_{t}\\ u_{2}/T_{t}\\ u_{3}/T_{t}\\ u_{1}^{2}\\ u_{1}u_{2}\\ u_{1}u_{3}\\ u_{2}u_{3}\\ u_{2}^{2}\\ u_{1}u_{1}^{\prime }\\ u_{2}u_{1}^{\prime }\\ u_{3}u_{1}^{\prime }\\ u_{1}u_{3}^{\prime }\\ u_{2}u_{3}^{\prime }\\ u_{3}u_{3}^{\prime }\\ u_{1}u_{2}^{\prime }\\ u_{3}u_{2}^{\prime } \end{array}\right],$$

where *b*_1_, *b*_2_, …, *b*_16_ are 16 compensation coefficients.

From the T-L method described above, it is apparent that $H_{t}$ is correlated with three components of the magnetic flux gate data, including their cosine and derivative values. Therefore, based on the T-L model, these parameters are selected as the input features of the neural networks. This paper does not require solving for T-L coefficients, but instead uses the neural network to find appropriate weights that transform input data into a correction that minimizes the noise (standard deviation) of the observed total field. We note that due to the lower measurement accuracy of triaxial fluxgate magnetometers compared to optically pumped magnetometers, the input three-component geomagnetic field data is calculated from the normal geomagnetic field and attitude angle information.

### 2.2. MLP Neural Networks

#### 2.2.1. Multilayer Perceptron

**Figure 2:**
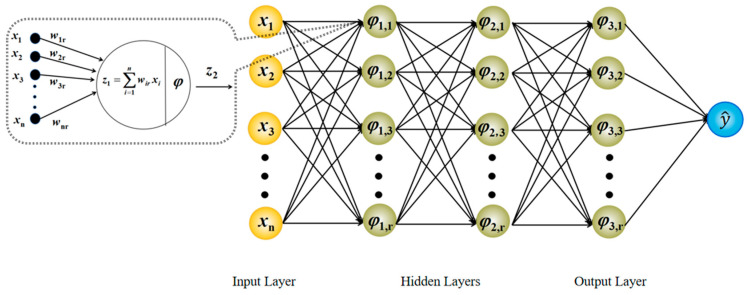
MLP neural networks model diagram.

The Multilayer Perceptron (MLP) is a traditional deep learning method based on the feedforward neural network architecture (as shown in Figure 2), consisting of three parts: the input layer, the hidden layer, and the output layer. The training of an MLP primarily relies on the backpropagation algorithm. Computing the gradient of the loss function of the network’s parameters and using gradient descent optimization, the weights are iteratively updated to minimize prediction errors.

#### 2.2.2. Forward Propagation

Forward propagation is used to compute the output of neural networks. In MLP, the input to the first hidden layer comes from the input layer. The computation for this layer is as follows:

(14) $$z^{\left[1\right]}=w^{\left[1\right]}x+b^{\left[1\right]}$$

(15) $$a^{\left[1\right]}=f\left(z^{\left[1\right]}\right)$$

where $z^{\left[1\right]}$ is the linear transformation result, $w^{\left[1\right]}$ is the first layer’s weight matrix, $b^{\left[1\right]}$ is the bias, and $a^{\left[1\right]}$ is the output of the activation function.

The calculation for the l-th layer (where l ranges from 2 to *L* − 1, and *L* is the total number of layers) is as follows:

(16) $$z^{\left[l\right]}=w^{\left[l\right]}a^{\left[l-1\right]}+b^{\left[l\right]}$$

(17) $$a^{\left[l\right]}=f\left(z^{\left[l\right]}\right)$$

where $z^{\left[l\right]}$ is the linear transformation result, $w^{\left[l\right]}$ is the *l*-th layer’s weight matrix.

The calculation expression of the output layer is as follows:

(18) $$z^{\left[L\right]}=w^{\left[L\right]}a^{\left[L-1\right]}+b^{\left[L\right]}$$

(19) $$\hat{y}=g\left(z^{\left[L\right]}\right)$$

where $z^{\left[L\right]}$ is the output result, $w^{\left[L\right]}$ is the output layer’s weight matrix.

In this paper, the Mean Squared error (MSE) is adopted to define the loss function as follows:

(20) $$Loss=\frac{1}{N}\sum_{i=1}^{N}{\left(y_{i}-{\hat{y}}_{i}\right)}^{2}$$

#### 2.2.3. Backpropagation

The goal of backpropagation is to compute gradients to update the neural network’s weights, minimizing the loss function. First, the output layer error is calculated by taking the derivative of the Loss with respect to $z^{\left[L\right]}$ as follows:

(21) $$\delta^{\left[L\right]}=\frac{\partial Loss}{\partial z^{\left[L\right]}}=\hat{y}-y$$

(22) $$a^{\left[1\right]}=f\left(z^{\left[1\right]}\right)$$

Subsequently, the error propagates from the output layer to the hidden layers. The hidden layer error is calculated as follows:

(23) $$\delta^{\left[l\right]}={\left(w^{\left[l+1\right]}\right)}^{T}\delta^{\left[l+1\right]}\cdot f^{\prime }\left(z^{\left[l\right]}\right)$$

(24) $$a^{\left[l\right]}=f\left(z^{\left[l\right]}\right)$$

Compute the gradients:

(25) $$\frac{\partial Loss}{\partial w^{\left[l\right]}}=\delta^{\left[l\right]}{\left(a^{\left[l-1\right]}\right)}^{T}$$

(26) $$\frac{\partial Loss}{\partial b^{\left[l\right]}}=\sum \delta^{\left[l\right]}$$

Update the parameters (e.g., Adam, SGD):

(27) $$w^{\left[l\right]\prime }=w^{\left[l\right]}-\alpha \frac{\partial Loss}{\partial w^{\left[l\right]}}$$

(28) $$b^{\left[l\right]\prime }=b^{\left[l\right]}-\alpha \frac{\partial Loss}{\partial b^{\left[l\right]}}$$

Before training, all weights and biases need to be randomly initialized. During the training process, multiple iterations are required until the network converges, ultimately obtaining the optimal model.

### 2.3. Transformer Neural Networks

**Figure 3:**
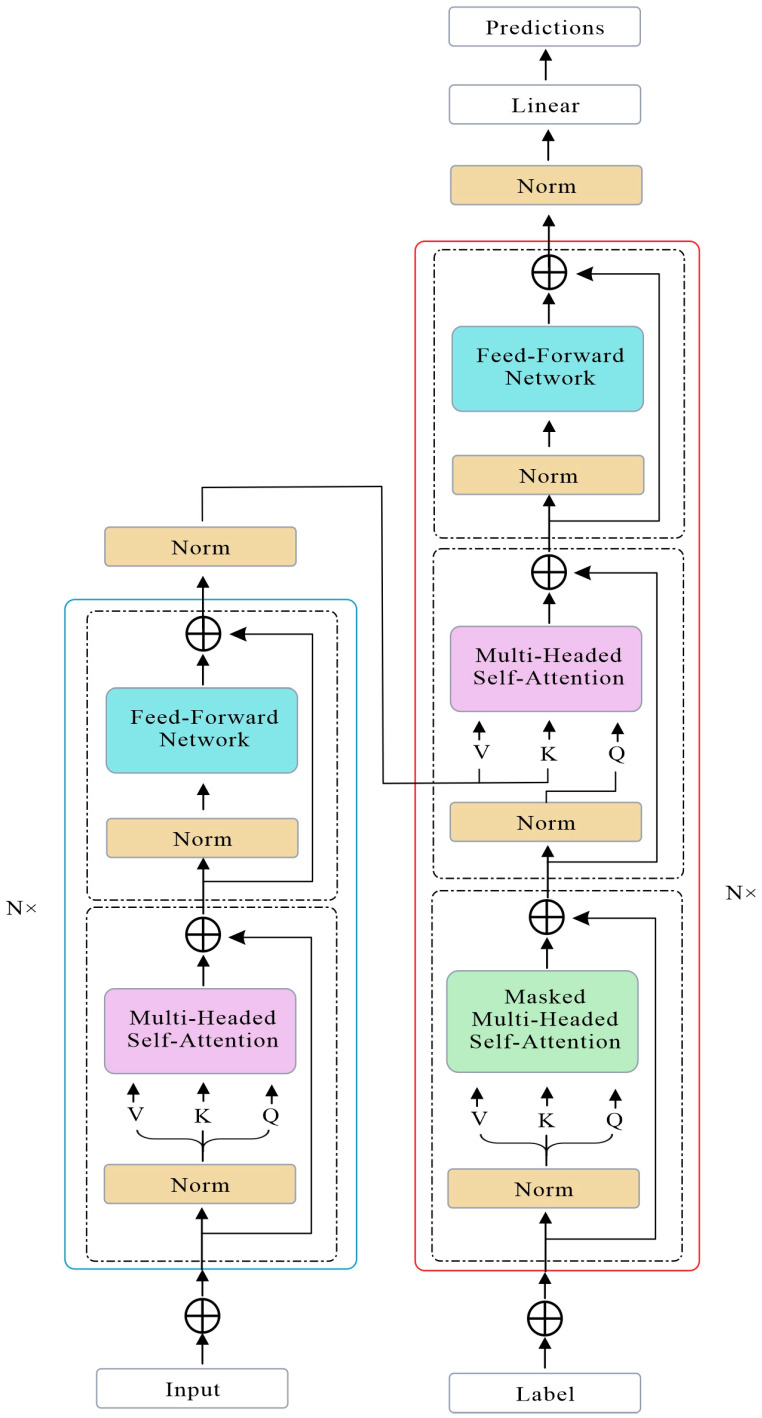
Transformer model diagram.

Transformer is a deep learning model based on a self-attention mechanism, and its main structure consists of an encoder and a decoder. Residual connections and layer normalization stabilize training. Transformers excel at capturing long-range dependencies and have become the foundation for state-of-the-art NLP models like BERT and GPT. Its model architecture is shown in Figure 3.

#### 2.3.1. Forward Propagation

The core of the self-attention mechanism lies in computing attention weights for the input sequence and aggregating information based on these weights. First, calculate the Query (Q), Key (K), and Value (V) matrices. The input $X\in R^{n\times d}$ is multiplied by learnable weight matrices $W_{Q}$, $W_{K}$, and $W_{V}$ respectively.

(29) $$Q=XW_{Q},\;K=XW_{K},\;V=XW_{V}$$

where Q represents the current position requiring attention computation, K serves to calculate similarity with other positions, and V contains the actual information used for weighted aggregation.

Computing the dot product between Q and K, and scaling by $\sqrt{d_{k}}$ to prevent the problem of gradient vanishing or explosion, and then used to calculate the attention weights.

(30) $$A=Softmax\left(\frac{QK^{T}}{\sqrt{d_{k}}}\right)$$

where A represents the attention weights from each position to all other positions.

Then computing weighted sum:

(31) S=AV

A linear transformation is applied using a learnable weight matrix $W_{O}\in R^{d_{k}\times d}$.

(32) $$O=SW_{O}$$

Moreover, a residual connection structure is incorporated, which enables direct gradient flow during backpropagation, effectively mitigating the vanishing gradient problem. Layer normalization (LayerNorm) is then applied to each sample to stabilize the training process.

$$X_{attn}=LayerNorm\left(X+O\right)$$

Next, the output is processed through an FFN layer. Typically, the FFN consists of a two-layer fully connected network, which enhances the model’s nonlinear expressive capability. Finally, another residual connection is applied to obtain the final output.

(33) $$H=ReLU\left(X_{attn}W_{1}+b_{1}\right)$$

(34) $$F=HW_{2}+b_{2}$$

(35) $$X_{out}=LayerNorm\left(X_{attn}+F\right)$$

where ReLU serves as the activation function; $W_{1}$ and $W_{2}$ are the weight matrices of the FFN layer, while $b_{1}$ and $b_{2}$ represent the bias terms.

#### 2.3.2. Backpropagation

Firstly, Self-Attention Layer gradient calculation. The mathematical formulation of LayerNorm is expressed as follows:

(36) $$X_{attn}=\gamma ⊙\frac{X+O-\mu }{\sigma }+\beta$$

where μ is the mean of X+O, σ is the standard deviation, and γ and β are the learnable scaling and shifting parameters, respectively.

Let the loss function be denoted as L, and the gradient of L with respect to $X_{attn}$ be denoted as $\frac{\partial L}{\partial X_{attn}}$. Then we obtain the following:

(37) $$\frac{\partial L}{\partial \left(X+O\right)}=\frac{\partial L}{\partial X_{attn}}⊙\gamma \cdot \left(\frac{1}{\sigma }-\frac{{\left(X+O-\mu \right)}^{2}}{n\sigma^{3}}\right)$$

For the gradients of the residual connection and output projection, since $X_{attn}=LayerNorm\left(X+O\right)$, it follows that:

(38) $$\frac{\partial L}{\partial O}=\frac{\partial L}{\partial \left(X+O\right)}$$

(39) $$\frac{\partial L}{\partial X}=\frac{\partial L}{\partial \left(X+O\right)}$$

Moreover, since $O=SW_{O}$, we obtain:

(40) $$\frac{\partial L}{\partial W_{O}}=S^{T}\frac{\partial L}{\partial O}$$

(41) $$\frac{\partial L}{\partial S}=\frac{\partial L}{\partial O}W_{O}^{T}$$

For gradient of attention weights, since S=AV, it follows that:

(42) $$\frac{\partial L}{\partial A}=\frac{\partial L}{\partial S}V^{T}$$

(43) $$\frac{\partial L}{\partial \left(QK^{T}/\sqrt{d_{k}}\right)}=\frac{\partial L}{\partial A}⊙\left(diag\left(A\right)-AA^{T}\right)$$

For gradient of, since $A=Softmax\left(\frac{QK^{T}}{\sqrt{d_{k}}}\right)$, we obtain:

(44) $$\frac{\partial L}{\partial Q}=\frac{1}{\sqrt{d_{k}}}\frac{\partial L}{\partial \left(QK^{T}/\sqrt{d_{k}}\right)}K$$

(45) $$\frac{\partial L}{\partial K}=\frac{1}{\sqrt{d_{k}}}{\left(\frac{\partial L}{\partial \left(QK^{T}/\sqrt{d_{k}}\right)}\right)}^{T}Q$$

(46) $$\frac{\partial L}{\partial V}=A^{T}\frac{\partial L}{\partial S}$$

Thus, the gradients of $W_{Q}$,$W_{K}$, and $W_{V}$ are as follows:

(47) $$\frac{\partial L}{\partial W_{Q}}=X^{T}\frac{\partial L}{\partial Q},\;\frac{\partial L}{\partial W_{K}}=X^{T}\frac{\partial L}{\partial K},\;\frac{\partial L}{\partial W_{V}}=X^{T}\frac{\partial L}{\partial V}$$

Secondly, compute the gradients for the FFN layer. The gradients for weight matrix $W_{2}$ and bias term $b_{2}$ are given by the following:

(48) $$\frac{\partial L}{\partial W_{2}}=H^{T}\frac{\partial L}{\partial F}$$

(49) $$\frac{\partial L}{\partial b_{2}}=\sum \frac{\partial L}{\partial F}$$

Since $H=ReLU\left(X_{attn}W_{1}+b_{1}\right)$, we obtain:

(50) $$\frac{\partial L}{\partial H}=\frac{\partial L}{\partial F}W_{2}^{T}$$

(51) $$\frac{\partial L}{\partial \left(X_{attn}W_{1}+b_{1}\right)}=\frac{\partial L}{\partial H}⊙{ReLU}^{\prime }\left(X_{attn}W_{1}+b_{1}\right)$$

The gradients of the weight $W_{1}$ and biase $b_{1}$ are calculated as follows:

(52) $$\frac{\partial L}{\partial W_{1}}=X_{attn}^{T}\frac{\partial L}{\partial \left(X_{attn}W_{1}+b_{1}\right)}$$

(53) $$\frac{\partial L}{\partial b_{1}}=\sum \frac{\partial L}{\partial \left(X_{attn}W_{1}+b_{1}\right)}$$

Thus, the gradients of $X_{attn}$ is as follows:

(54) $$\frac{\partial L}{\partial X_{attn}}=\frac{\partial L}{\partial F}+\frac{\partial L}{\partial \left(X_{attn}W_{1}+b_{1}\right)}W_{1}^{T}$$

Thirdly, computing gradients for LayerNorm Parameters. The gradients of γ and β are expressed as follows:

(55) $$\frac{\partial L}{\partial \gamma }=\sum_{i=1}^{n}\frac{\partial L}{\partial X_{attn}^{i}}⊙{\hat{X}}^{i}$$

(56) $$\frac{\partial L}{\partial \beta }=\sum_{i=1}^{n}\frac{\partial L}{\partial X_{attn}^{i}}$$

where ${\hat{X}}^{i}$ is the normalized result of the i-th sample.

Finally, gradient descent is applied to update the parameters. For example, the updated parameters θ is computed as follows:

(57) $$\theta^{\prime }=\theta -\eta \frac{\partial L}{\partial \theta }$$

where η is learning rate, $\theta \in (W_{Q},W_{K},W_{V},W_{O},W_{1},b_{1},W_{2},b_{2},\;\gamma ,\beta ).$

## 3. Simulation and Compensation

### 3.1. Experiment of Simulation

In actual aeromagnetic surveys, aeromagnetic compensation requires standardized flight maneuvers. This standardized flight method, known as the FOM (Figure of Merit Flight), designed by Leliak [30]. The FOM flight needs the aircraft to fly along predetermined directions. In each direction, it performs three sets of maneuvers: pitch, roll, and yaw. Each maneuver lasts approximately 5~10 s, with 5 s of level flight inserted between sets. With the development of drones, an alternative approach has emerged: drones can perform in-place motion to achieve the same effect as FOM flight during actual measurements.

**Figure 4:**
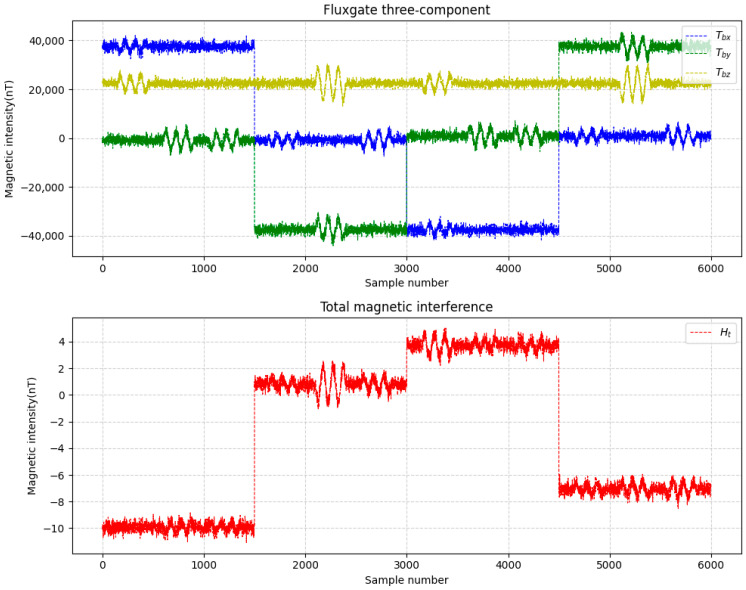
Data A.

**Figure 5:**
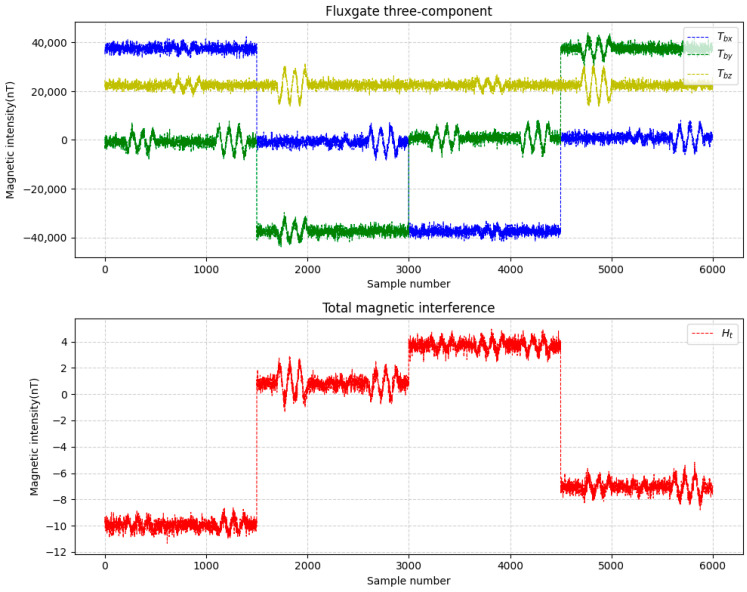
Data B.

This study simulates the attitude variations in a drone and configures 16 compensation coefficients, parameters such as attitude angles, the magnetization bias angle, the magnetization tilt angle et al. Meanwhile, appropriate noise was added during data simulation. Two datasets were generated and labeled as Data A and Data B. The three-axis fluxgate magnetometer components ($T_{bx}$, $T_{by}$, $T_{bz}$) and the total magnetic interference ($H_{t}$) for these datasets are illustrated in Figure 4 and Figure 5.

### 3.2. Compensation Results

This paper selects standard deviation (STD) and improvement ratio (IR) to evaluate the effect of aeromagnetic compensation, and then compares and analyzes the effectiveness of the methods.

(58) $$STD=\sqrt{\frac{1}{n}\sum_{i=1}^{n}\left(\mu -\bar{\mu }\right)}$$

where n is the sample number, μ represents the sample value, and $\bar{\mu }$ is the mean of μ.

The improvement ratio is calculated by *STD*s as follows:

(59) $$IR=\frac{STD_{u}}{STD_{c}}$$

where $STD_{u}$ and $STD_{c}$ are the uncompensated magnetic data and compensated data, respectively.

**Figure 6:**
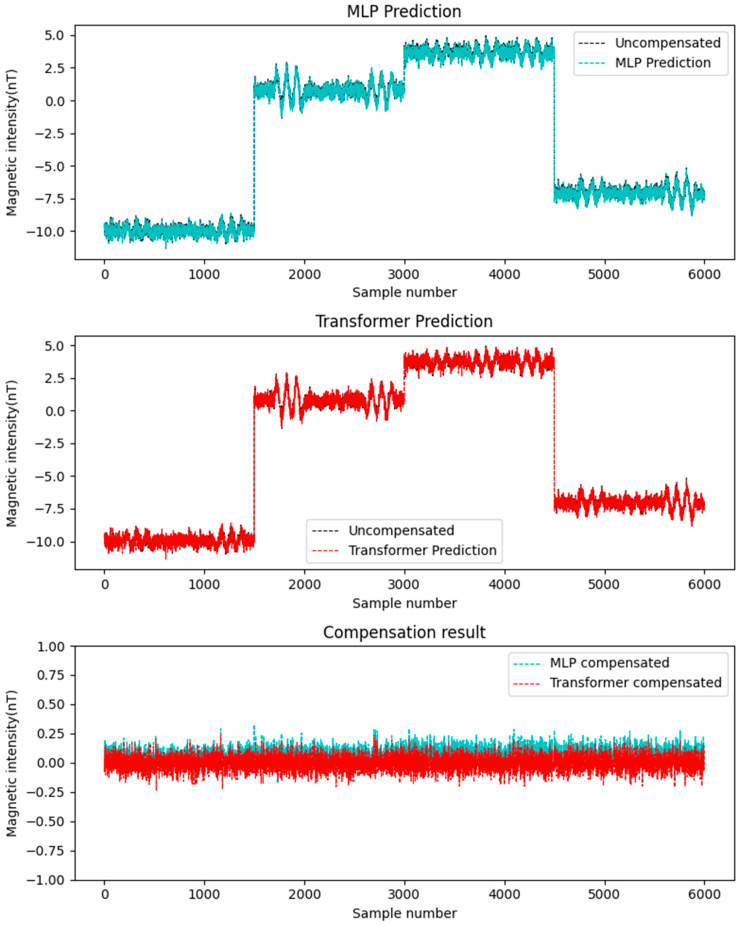
Data A compensation result.

**Figure 7:**
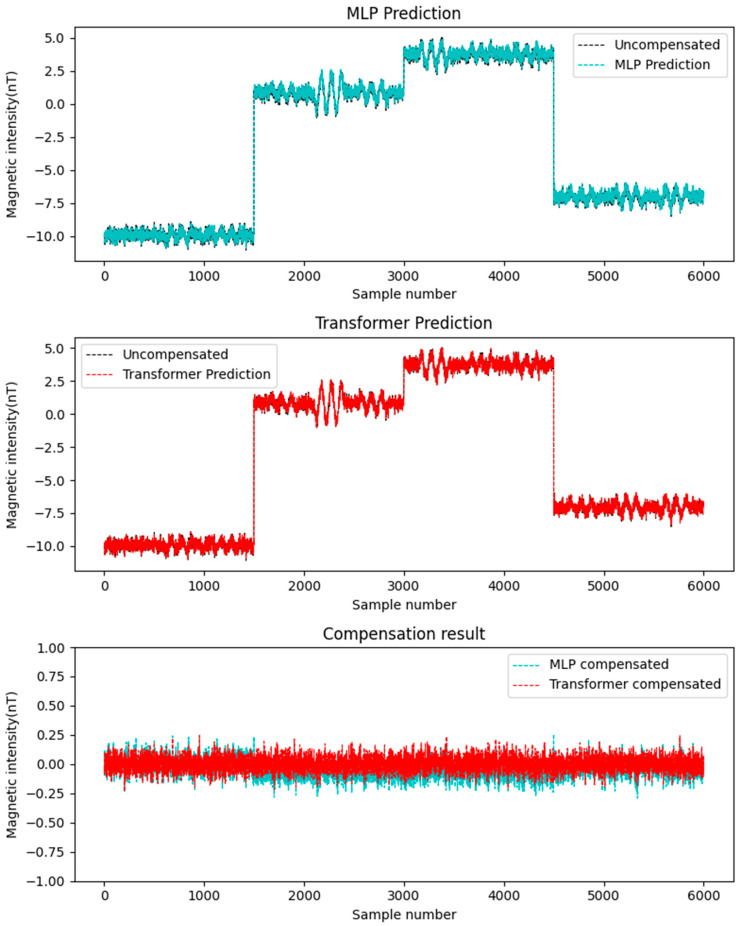
Data B compensation result.

In this study, the simulated datasets Data A and Data B were alternately used as the training set and test set. Considering that the compensation accuracy of traditional linear methods (such as least squares method, LS) is not as good as that of neural network methods [23], this paper adopts MLP to compare with Transformer. The aforementioned two neural networks were employed for compensation, with the results depicted in Figure 6 and Figure 7. The figures demonstrate the following: First, neural network-based compensation achieves significant improvement. Second, the Transformer model outperforms MLP in compensation effectiveness. Meanwhile, a quantitative comparison is provided in Table 1, showing that: For Transformer, the standard deviation of compensated data is smaller than MLP, and the improvement rate is higher. These results confirm the better performance of the Transformer networks for compensation tasks on simulated data.

## 4. Field-Measured Data Compensation

### 4.1. Flight of Compensation

**Figure 8:**
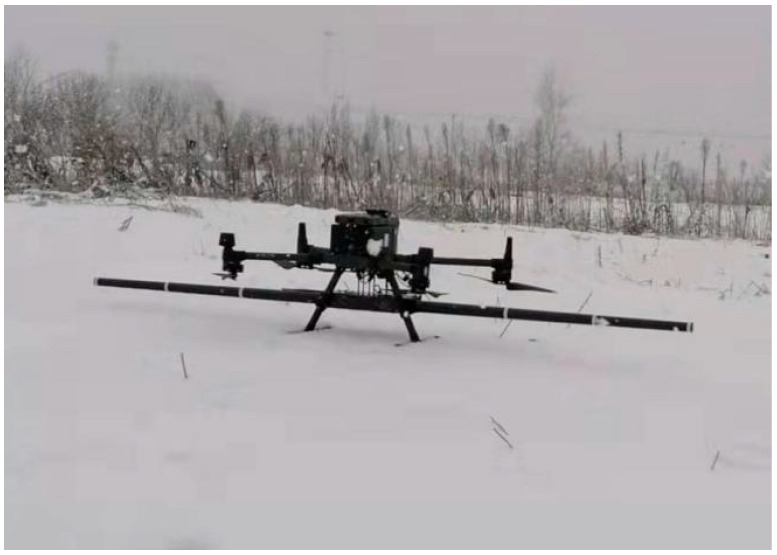
Unmanned aerial vehicles and equipment.

To meet the hypothetical conditions of the T-L model, this study conducted FOM flights using a DJI M300RTK quadcopter UAV equipped with an airborne magnetic survey system (as shown in Figure 8) in a designated area of Suqian City, Jiangsu Province, China. Here we employ a miniature rubidium optically pumped magnetometer, with its main performance parameters shown in Table 2.

**Figure 9:**
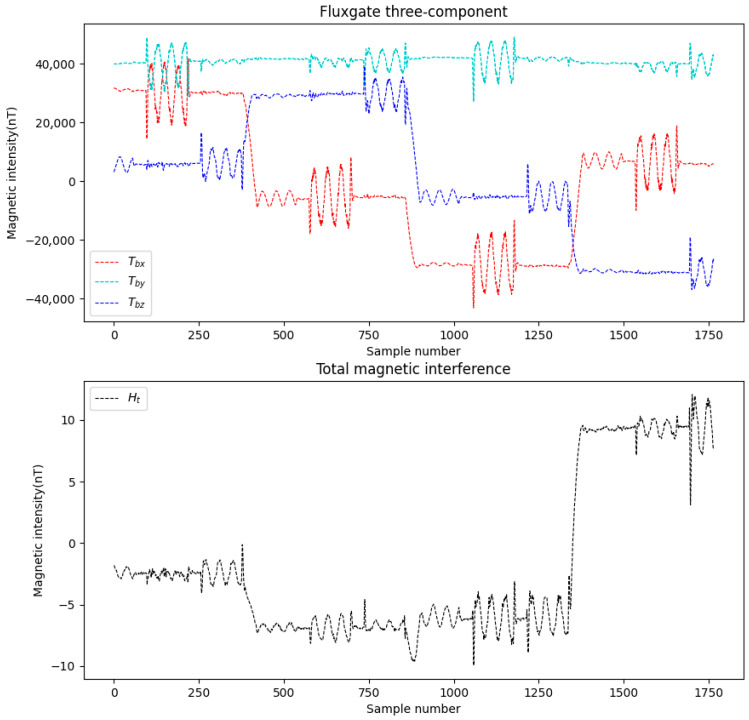
Data C.

**Figure 10:**
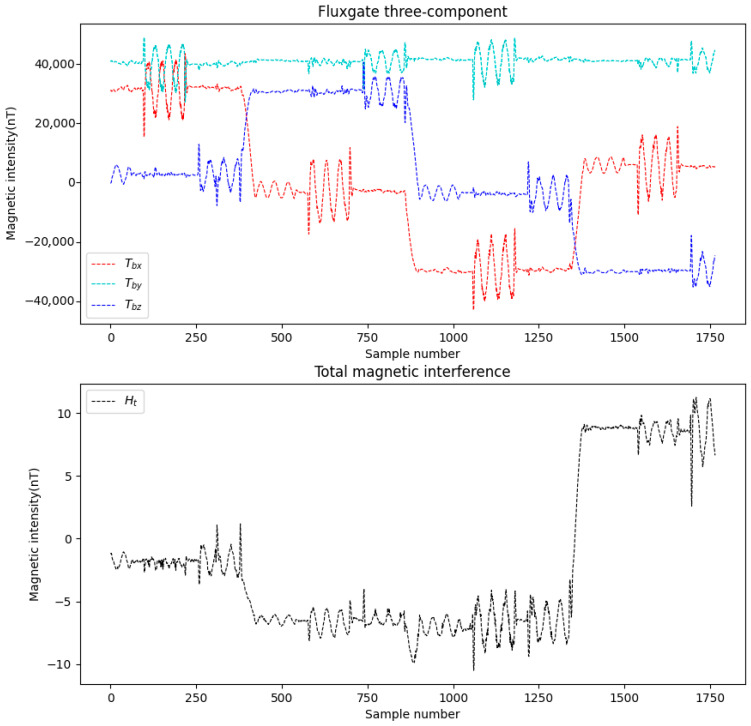
Data D.

Two FOM flights were conducted in this study, which selected sites with features such as flat terrain, minimal geomagnetic variations, and an absence of interference sources (e.g., high-voltage power lines) and acquired two datasets labeled as Data C and Data D, shown in Figure 9 and Figure 10.

### 4.2. Compensation Results

**Figure 11:**
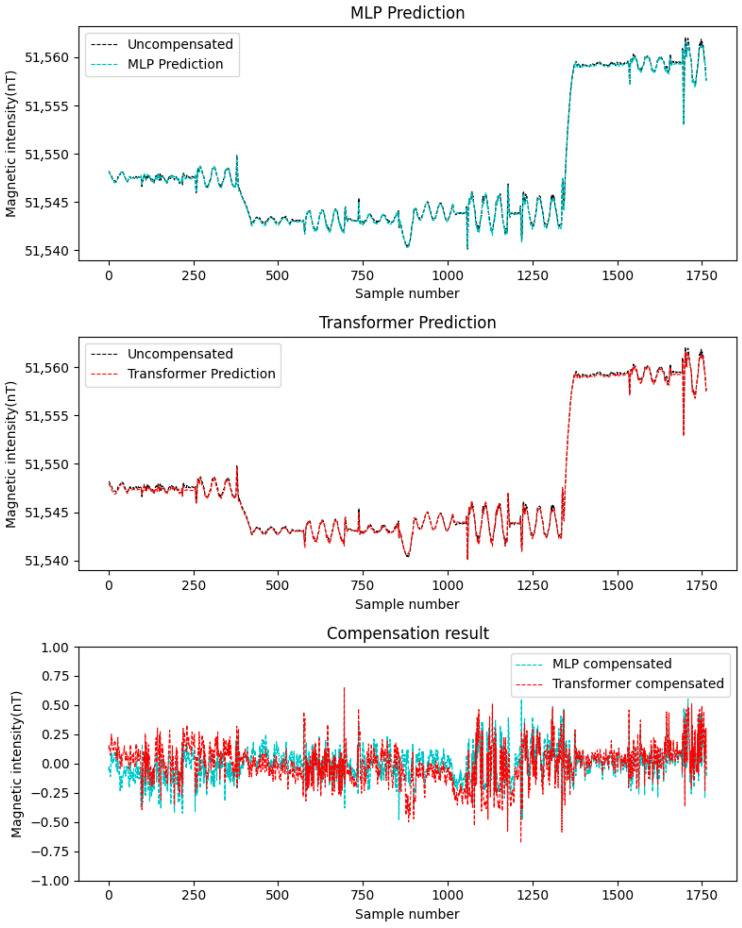
Data C compensation result.

**Figure 12:**
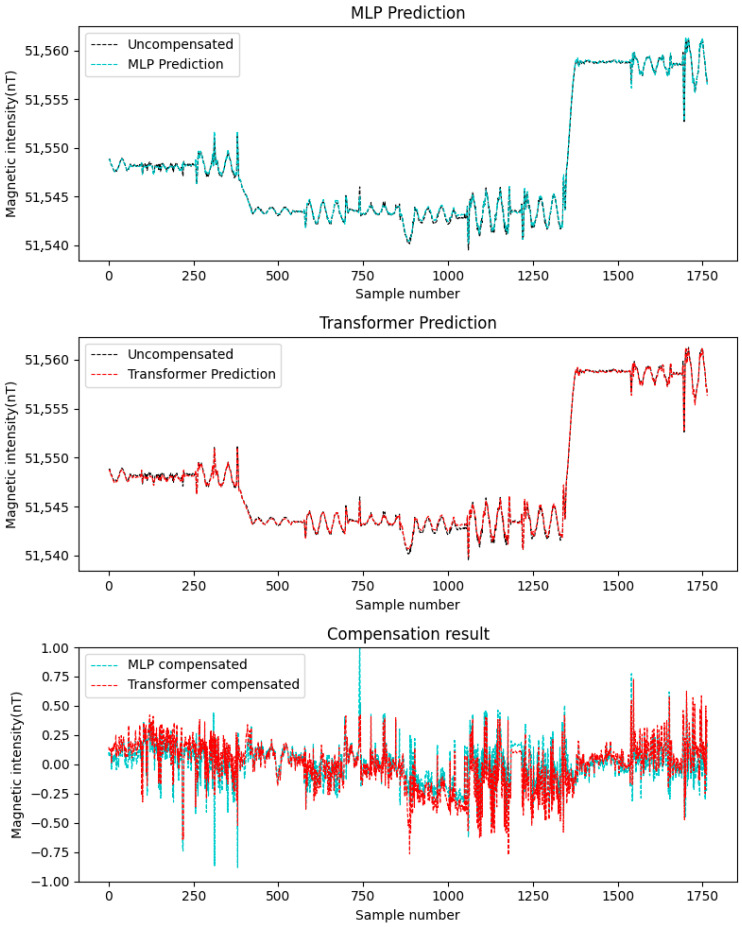
Data D compensation result.

Similarly, the measured datasets Data C and Data D were alternately used as the training set and test set. The aforementioned two neural networks were employed for compensation, with the results depicted in Figure 11 and Figure 12. Meanwhile, a quantitative comparison is provided in Table 3. These results also confirm the better performance of the Transformer neural networks for compensation tasks on measured data.

## 5. Conclusions

In conventional aeromagnetic compensation tasks, linear regression algorithms have traditionally dominated. While the standard MLP neural network exhibits stronger fitting capabilities than linear regression and offers some improvement in compensation accuracy, it remains a global approximation neural network with limited generalization ability. Issues such as gradient vanishing further constrain its precision gains for aeromagnetic compensation. To enhance compensation accuracy, this study builds upon the T-L model and proposes a Transformer neural network-based aeromagnetic compensation method. The approach captures temporal dependencies in the data, further refining compensation precision. The feasibility of this method was validated through experiments on simulated and measured flight data. The development of high-precision aeromagnetic compensation methods also provides important support for the application of unmanned aerial vehicle aeromagnetic measurement technology in fields such as metal mine detection and military target detection in the later stage.

## Figures and Tables

**Table 1:** Compensation results of the simulation data.

Test Set	Training Set	Method	${STD}_{p}$	${STD}_{f}$	IR
Data A	Data B	MLP	5.59	0.069	82.45
		Transformer		0.065	86.40
Data B	Data A	MLP	5.58	0.074	75.38
		Transformer		0.065	86.38

**Table 2:** Parameters of miniature rubidium optically pumped magnetometer.

Measuring Range	1000~100,000 nT
Gradient Capacity	100 nT/cm
Ground Static Noise Level	≤0.01 nT
Resolution	0.0001 nT
Sensitivity	0.02 nT/$\sqrt{\mathrm{Hz}}$
Power Consumption	≤3 W
Operating Remperature	−30~+60 °C

**Table 3:** Simulation model parameters.

Test Set	Training Set	Method	${STD}_{p}$	${STD}_{f}$	IR
Data D	Data C	MLP	6.21	0.17	31.13
		Transformer		0.13	36.43
Data C	Data D	MLP	6.42	0.16	40.94
		Transformer		0.14	46.57

## Data Availability

The original contributions presented in this study are included in the article. Further inquiries can be directed to the corresponding author.
